# Incorporating breeding abundance into spatial assignments on continuous surfaces

**DOI:** 10.1002/ece3.2605

**Published:** 2017-04-21

**Authors:** Clark S. Rushing, Peter P. Marra, Colin E. Studds

**Affiliations:** ^1^ Migratory Bird CenterSmithsonian Conservation Biology InstituteNational Zoological ParkWashingtonDCUSA; ^2^ Department of Geography & Environmental SystemsUniversity of Maryland Baltimore CountyBaltimoreMDUSA

**Keywords:** Bayes rule, migratory connectivity, Neotropical migratory birds, Pareto optimality, probabilistic assignment, stable‐hydrogen isotopes

## Abstract

Determining the geographic connections between breeding and nonbreeding populations, termed migratory connectivity, is critical to advancing our understanding of the ecology and conservation of migratory species. Assignment models based on stable isotopes historically have been an important tool for studying migratory connectivity of small‐bodied species, but the low resolution of these assignments has generated interest into combining isotopes with other sources in information. Abundance is one of the most appealing data sources to include in isotope‐based assignments, but there are currently no statistical methods or guidelines for optimizing the contribution of stable isotopes and abundance for inferring migratory connectivity. Using known‐origin stable‐hydrogen isotope samples of six Neotropical migratory bird species, we rigorously assessed the performance of assignment models that differentially weight the contribution of the isotope and abundance data. For two species with adequate sample sizes, we used Pareto optimality to determine the set of models that simultaneously minimized both assignment error rate and assignment area. We then assessed the ability of the top models from these two species to improve assignments of the remaining four species compared to assignments based on isotopes alone. We show that the increased precision of models that include abundance is often offset by a large increase in assignment error. However, models that optimally weigh the abundance data relative to the isotope data can result in higher precision and, in some cases, lower error than models based on isotopes alone. The top models, however, depended on the distribution of relative breeding abundance, with patchier distributions requiring stronger downweighting of abundance, and we present general guidelines for future studies. These results confirm that breeding abundance can be an important source of information for studies investigating broad‐scale movements of migratory birds and potentially other taxa.

## 1 Introduction

Understanding how migratory species redistribute themselves across the annual cycle, known as migratory connectivity, is essential for understanding range dynamics, identifying key threats, and developing coordinated conservation actions. Satellite tracking has revolutionized migratory connectivity research for large‐bodied species (>100 g) by enabling remote transmission of individual movements over broad spatial and temporal scales (Block et al., 2011). For species too small to carry these transmitters, morphological and chemical signatures of individual organisms or tissues, termed intrinsic markers, are essential tools for estimating migratory connectivity.

Stable isotopes are arguably the most useful intrinsic marker for studying migratory connectivity because of their comparatively low cost and scale of inference (Hobson, 2008) and have supported important advances for numerous taxa, including birds (Rubenstein & Hobson, 2004), mammals (Sullivan, Bump, Kruger, & Peterson, 2012), and insects (Hobson, Soto, Paulson, Wassenaar, & Matthews, 2012; Hobson, Van Wilgenburg, Wassenaar, & Larson, 2012). Although stable isotopes offer lower spatial resolution compared to direct tracking, substantial progress has occurred by combining stable isotopes with other intrinsic markers such as genetic data (Kelly, Ruegg, & Smith, 2005; Rundel et al. 2013), morphometrics (Rushing, Ryder, Saracco, & Marra, 2014), and band recoveries (Hobson, Wunder, Van Wilgenburg, Clark, & Wassenaar, 2009; Van Wilgenburg & Hobson, 2011), or with information on abundance (Flockhart et al., 2013; Hallworth, Studds, Sillett, & Marra, 2013).

Abundance is one of the most appealing data sources to include in migratory connectivity analyses because high‐quality, range‐wide data are often freely available. However, a recently noted problem with combing stable isotopes and abundance in migratory connectivity estimates is that many species have patchy breeding distributions. This causes centers of high abundance to be overrepresented and areas of low abundance to be underrepresented in assignments to origin (Hobson et al., 2014). Despite their wide use, there is no analytical method and no guidelines for optimizing the contribution of stable isotopes and abundance for inferring migratory connectivity.

The first formal method to include breeding abundance and intrinsic markers in geographic assignments was outlined by Royle and Rubenstein (2004). In their model, stable isotopes are used to assign individuals to one of a finite number of discrete breeding populations, denoted by *b* = 1, 2, … *B*. Each population is defined by a probability distribution that describes the expected values of the marker for individuals originating in that population. From this distribution, it is straightforward to estimate the likelihood that each population is the origin of an individual with an observed marker value y*, denoted *f*(y*|*b*). Because areas differ with regard to relative abundance, it may be reasonable to assume that individuals are more likely to originate from high abundance populations than low abundance populations. Using Bayes rule, the relative abundance can be incorporated into the assignment model as a prior probability. This allows researchers to explicitly model the probability that an individual originated from each population, that is, *f*(*b*|y*), and to formally base assignments on both the isotope data (via the likelihood) and breeding abundance (via the prior).

Although the original model outlined by Royle and Rubenstein (2004) was developed to make geographic assignments to a few discrete breeding regions, the increasing availability of global isoscape and abundance surfaces has enabled researchers to make assignments on nearly continuous landscapes (Hobson et al., 2009; Sullivan et al., 2012; Van Wilgenburg & Hobson, 2011)**.** This introduces a critical complication that has gone largely unrecognized. The problem arises because most species are patchily distributed across their range, with a few areas of relatively high abundance and extensive areas of lower abundance. When abundance is included as a prior in assignments to continuous surfaces, the low abundance sites may receive low posterior support compared to high abundance locations. Although this outcome is consistent with the logic proposed by Royle and Rubenstein (2004), in practice, the posterior probabilities for each location may simply reflect relative abundance, thus limiting the contribution of the isotope data to assignments (see González‐Prieto, Hobson, Bayly, & Gómez, 2011; Hobson et al., 2014). In extreme cases, the inclusion of breeding abundance may lead to inaccurate assignments and obscure estimates of migratory connectivity, the biological processes of interest.

In this study, we describe a new quantitative method for making geographic assignments to origin that differentially weights stable‐hydrogen isotope and breeding abundance data to maximize assignment area and minimize assignment error. We performed Bayesian assignments of origin for six species of Neotropical‐Nearctic migratory birds: Wood Thrush (*Hylocichla mustelina*), Northern Parula (*Setophaga americana*), Prairie Warbler (*Setophaga discolor*), Black‐and‐White Warbler (*Mniotilta varia*), American Redstart (*Setophaga ruticilla*), and Ovenbird (*Seiurus aurocapilla*). Using stable‐hydrogen isotope data collected at known breeding sites across the breeding range of each species enabled us to assess the performance of different models. Using Pareto optimality, a method for multi‐objective optimization, we show that weighting the isotope and abundance data can increase the performance of assignment models but that the distribution of breeding abundance plays a critical role in determining the proper weightings.

## 2 Materials and Methods

### 2.1 Study species and feather sampling

To determine whether the inclusion of abundance data improved the performance of assignment models that rely on isotope data alone, we used stable‐hydrogen isotope data from six species of Neotropical migratory birds collected at known breeding locations (Tables 1 and S1–S6). The use of known‐origin samples allowed us to test explicitly the performance of alternative assignment models. Feather samples were collected from 2009 to 2011 at six breeding sites in the eastern United States that span the geographic extent of the breeding range and include a wide range of breeding abundance for each species. Vegetation types included bottomland hardwoods, coastal plain forest, northern hardwoods, and spruce‐fir forest at elevations from 5 to 1,000 m. Birds were captured using mist nets, aged and sexed using criteria from Pyle (1997), banded with a United States Geological Survey (USGS) aluminum leg band, and released. One tail feather was removed from each bird before release and stored in a paper envelope. Stable‐hydrogen isotope values in feathers grown on or near breeding sites are strongly correlated with stable‐hydrogen isotope values in growing‐season precipitation and therefore provide information about breeding origin (Hobson, & Wassenaar, 1997). Each of the six species molts their tail feathers following reproduction, usually from late August to early September. We therefore restricted our analyses to adult tail feathers collected between 1 June and 31 July. We did not analyze feathers from immature birds because their isotope values can reflect natal origins.

**Table 1:** Summary of sampling data. Abundance range indicates the minimum and maximum predicted breeding abundance at the sampling locations for each species. For each species, the precipitation‐based hydrogen isoscape from Bowen et al. (2005) was converted to expected feather values using the slope parameter for long‐distance migrants from Van Wilgenburg, et al., 2012, with either the intercepts for ground or nonground foragers

Species	*n*	Abundance range (Maximum)^a^	Foraging height
Wood Thrush	120	3.26–17.89 (33.43)	Ground
American Redstart	110	0.05–7.05 (40.4)	Nonground
Ovenbird	30	0.02–22.22 (57.92)	Ground
Northern Parula	27	0.85–10.73 (25.22)	Nonground
Black‐and‐White Warbler	20	0.79–3.57 (12.51)	Nonground
Prairie Warbler	27	0.09–2.21 (23.35)	Nonground

a Abundance is expressed as the predicted number of birds per BBS route estimated through inverse distancing (Sauer et al., 2015).

Isotope analyses were performed at the Stable Isotope Mass Spectrometry Facility of the Smithsonian Institution in Suitland, MD. Feathers were washed in a 2:1 chloroform:methanol solution to remove surface oils and air‐dried under a fume hood for 48 hr. After transport to the laboratory, feathers were allowed to equilibrate with the local atmosphere for 72 hr. A small sample of each feather (0.30–0.35 mg) was packed into a silver capsule, combusted at 1,350°C in an elemental analyzer (Thermo TC/EA), and introduced online to an isotope ratio mass spectrometer (Thermo Delta V Advantage) via a Conflo IV interface. Four previously calibrated keratin standards were run for every 10 unknowns to account for exchangeable and nonexchangeable H in feather samples (IAEA‐CH‐7: δ^2^H = −100.3‰ Vienna standard mean ocean water [VSMOW]; Caribou Hoof Standard: δ^2^H = −197‰ VSMOW; Kudu Horn Standard: δ^2^H = −54.1 ‰ VSMOW; Spectrum Keratin Fine Powder: δ^2^H = −121.6 ‰ VSMOW). The δ^2^H values reported include only nonexchangeable H as determined by linear regression with the IAEA‐CH‐7, CHS, and KHS keratin standards (Wassenaar & Hobson, 2003) and are expressed in per mil units (‰) relative to the VSMOW‐Standard Light Antarctic Precipitation (VSMOW‐SLAP) scale. Replicate samples of the Spectrum Keratin Fine Powder standard and duplicate samples run for one in five to eight feathers indicated that analytical error (±1 *SD*) was <2‰.

Due to the comparatively small number of samples for Ovenbird, Northern Parula, Black‐and‐White Warbler, and Prairie Warbler, we restricted our full analysis of assignment models to Wood Thrush and American Redstart. After determining the top assignment models for those two species, we used the remaining four species for independent validations.

### 2.2 Stable isotope assignment model

To assign individuals to potential breeding locations, we first created base maps describing the variation in hydrogen isotope abundance and relative abundance across the breeding range of each species. To estimate the hydrogen isoscape, we converted a map of expected amount‐weighted growing‐season precipitation δ^2^H values ($\delta^{2}H_{p}$; Bowen et al. 2005) to expected feather δ^2^H values ($\delta^{2}H_{f}$) using published corrections for either ground foraging or nonground foraging long‐distance migratory birds (Hobson, Van Wilgenburg, et al., 2012). In their analysis, Hobson, Van Wilgenburg, et al., 2012 found no support for age‐based differences in hydrogen isotope discrimination, and therefore, we did not apply any age‐specific correction to the δ^2^H values.

Next, we assigned each bird to potential breeding locations using isotope values only. To do this, we calculated the likelihood that each raster cell represented the breeding location for each individual using a normal probability density function:

(1) $$f\left(y^{*}|\mu_{i},\sigma \right)=\frac{1}{\sqrt{2\pi \sigma }}\text{exp}\left[-\frac{1}{2\sigma^{2}}{\left(y^{*}-\mu_{i}\right)}^{2}\right]$$

where *f*(y*|μ_i_,σ) is the likelihood that an individual with $\delta^{2}H_{f}=y^{*}$ originated from cell *i*, μ_*i*_ is the predicted $\delta^{2}H_{f}$ value for cell *i,* and σ is the standard deviation of $\delta^{2}H_{f}$ values within a single breeding site, which was assumed to be 12 ‰ (Rushing et al., 2014). Next, we converted the likelihood values to a probability surface by dividing each likelihood by the sum of all of the likelihoods (Hobson et al., 2009; Van Wilgenburg & Hobson, 2011). We then sorted this probability surface from minimum to maximum and used a smoothing spline function to estimate the probability value (i.e., cutoff) that separated the upper 67% of the cumulative probabilities from the lower 33% (Chabot, Hobson, Van Wilgenburg, McQuat, & Lougheed, 2012; Hobson et al., 2009). Finally, we reclassified any cell with probability greater than the cutoff value as a likely (1) breeding origin and any cell with probability less than the cutoff as an unlikely (0) origin (Chabot et al., 2012; Hobson et al., 2009; Rushing et al., 2014). For each species and each sampling location, we then estimated the proportion of individuals that were misclassified (i.e., true breeding origin classified as unlikely; hereafter referred to as the error rate) and the mean proportion of raster cells classified as likely (referred to as the assignment area). Because the goal of assignments is to correctly classify the breeding locations while minimizing the assignment area, these two metrics provide quantitative and intuitive measures of model performance.

### 2.3 Incorporating abundance

**Figure 1:**
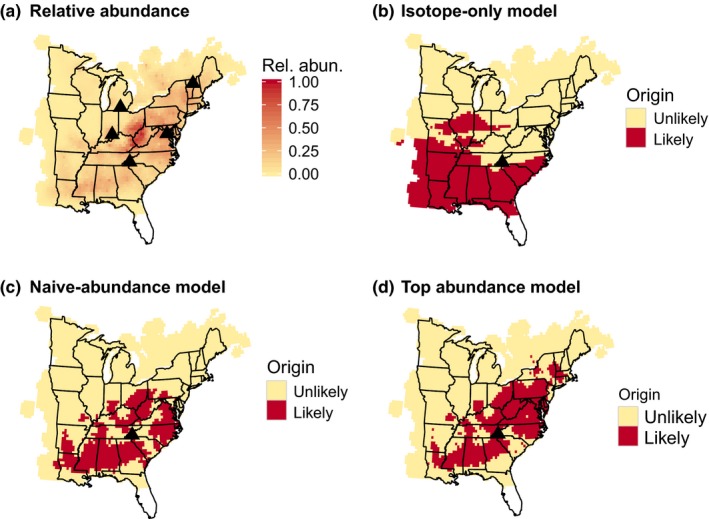
(a) Wood Thrush breeding abundance and sampling locations; (b) likely origins based on stable‐hydrogen isotopes for one individual originating in North Carolina; (c) likely origins based on unweighted isotope and breeding abundance (i.e., naive model) for the same individual; (d) likely origins based on the top Wood Thrush model (abundance weight = 10^0^, isotope weight = 10^−7^)

**Figure 2:**
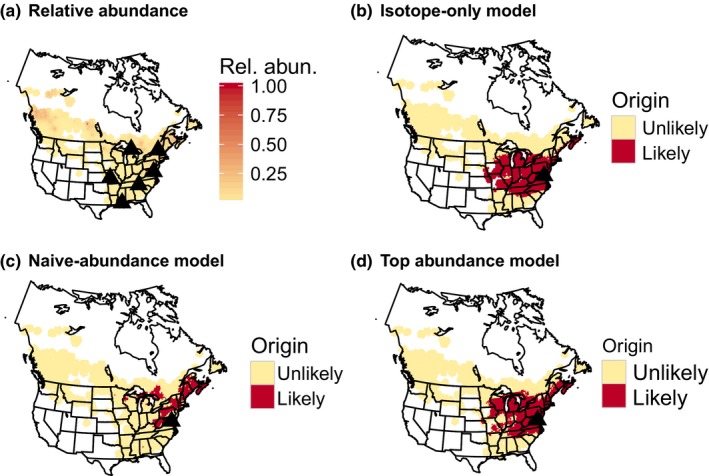
(a) American Redstart breeding abundance and sampling locations; (b) likely origins based on stable‐hydrogen isotopes for one individual originating in Maryland; (c) likely origins based on unweighted isotope and breeding abundance (i.e., naive model) for the same individual; (d) likely origins based on the top American Redstart model (abundance weight = 10^−1^, isotope weight = 10^0^). Under this model, the likely origins are still biased toward high abundance locations but lower abundance sites, including the true origin, still receive moderately high posterior support

Initially, we incorporated breeding abundance into the assignment model following the method outlined in recent assignment studies (Hallworth et al., 2013; Hobson et al., 2014). We used data from the North American Breeding Bird Survey (BBS) to create base maps of breeding abundance for each species (Figures 1a, 2a and S1; Sauer et al., 2015). Raw abundance estimates were then converted into a probability surface by dividing each cell by the sum of all cells (Hallworth et al., 2013). These relative abundance estimates were incorporated into the assignment model using Bayes rule:

(2) $$f\left(b_{i}|y^{*}\right)=\frac{f\left(y^{*}|b_{i}\right)f\left(b_{i}\right)}{\sum_{i=1}^{B}f\left(y^{*}|b_{i}\right)f\left(b_{i}\right)}$$

where *f*(*b*_*i*_|y*) is the posterior probability that an individual with δ^2^H_*f*_ = y* originated from cell *i, f*(y*|*b*_*i*_) is the likelihood of assignment to breeding cell *i*, and *f*(*b*_*i*_) is the relative abundance (i.e., the prior probability) of cell *i*. Because the abundance data in equation (2) were unweighted (see below), we refer to this model as the “naive‐abundance” model. As before, the posterior probabilities were converted to likely and unlikely origins using a 67% odds, and we quantified assignment performance using the assignment error rate and assignment area metrics described above.

### 2.4 Data weighting and model comparison

As described above, the combination of isotope data and abundance data described by equation (2) may be problematic if the prior probability imposed by the abundance data overwhelms the likelihood estimated from the isotope data. If this is the case, weighting the abundance and/or isotope data may be necessary to obtain unbiased estimates of geographic origins (Rundel et al., 2013). To determine whether weighting the two data sources improved the assignment model performance, we followed Rundel et al. (2013) and weighted the likelihood and abundance prior in equation (2) by raising each to all powers from 10^−1^ to 10, respectively, resulting in 442 assignment models (22^2^ = 441 abundance models + isotope‐only model). Powers >1 sharpen the distribution of values, giving more weight to high values relative to low values. Powers <1, in contrast, flatten the distribution and give more relative weight to low values. For each model, we estimated the assignment error and assignment area as described above.

After fitting the models for Wood Thrush and American Redstart, we used a two‐step approach to determine the top‐performing model(s). First, we used an optimization method termed Pareto optimality to eliminate from consideration any model that did not minimize both error rate and assignment area relative to other models. Pareto optimality is widely used in economics and engineering (Censor, 1977; Steuer, 1986) but has also been applied to a number of optimization problems in ecology and evolution (Kennedy, Ford, Singleton, Finney, & Agee, 2008; Reynolds & Ford, 1999; Shoval et al., 2012). Briefly, Pareto optimality describes a situation where a change in the system (e.g., changing the abundance and/or isotope weights) cannot improve one performance metric without worsening the other. Pareto optimal models are said to “dominate” all other models. For example, in this analysis, Pareto optimal models have both lower assignment area and error rate than all nonoptimal models in the set. The subset of models that are Pareto optimal form the Pareto frontier, along which one cannot improve assignment error without increasing assignment area and vice versa. By considering only models along the Pareto frontier, we were able to eliminate a large number of models from further consideration and to explicitly define the trade‐offs between assignment error and assignment area for only a few models.

Although identifying the Pareto frontier allowed us to restrict our attention to models that cannot be strictly improved with regard to both assignment area and assignment error, these models do not necessarily represent acceptable solutions for incorporating abundance into assignments. In particular, some models along the Pareto frontier may have large error rates and are thus inappropriate for assignment of unknown‐origin birds. Therefore, as a second step in determining the top model(s), we compared the assignment area and the error rate of each Pareto optimal model to the area and error rate of the isotope‐only model, under the assumption that any model that includes abundance should at least improve upon the isotope‐only model. Thus, the final model set included only Pareto optimal models that outperformed the isotope‐only model along both axes.

### 2.5 Sampling location and multispecies validation

One concern with our approach is that the top models for Wood Thrush and American Redstart may be specific to the sampling locations included in our analysis. To test whether our results were sensitive to the specific sampling locations included in the analysis, we iteratively removed all individuals from each site and re‐estimated the top models by comparing the new Pareto optimal models to the isotope‐only model (estimated using the same individuals). If the abundance and isotope weights remained constant across these scenarios, we concluded that the top models were robust to any differences in the sampling locations included in the analysis.

Species also vary considerably in their geographic distributions and patterns of abundance, and it is possible that these differences may influence the performance of assignment models based on isotopes and abundance. Unfortunately, sample sizes for four of the species included in our analysis (Ovenbird, Black‐and‐White Warbler, Northern Parula, and Prairie Warbler) were too small to obtain reliable estimates of top model weights using the Pareto method described above. Instead, we tested whether the top models identified for Wood Thrush and American Redstart outperformed assignments based on the isotope‐only model for the remaining species. For each species, we compared the assignment performance of the top Wood Thrush and American Redstart models to the performance of the respective isotope‐only model. If the top Wood Thrush and American Redstart models outperformed the isotope‐only models for the other species, we concluded that those models provide a general solution for assignments of Neotropical migratory songbirds.

## 3 Results

**Figure 3:**
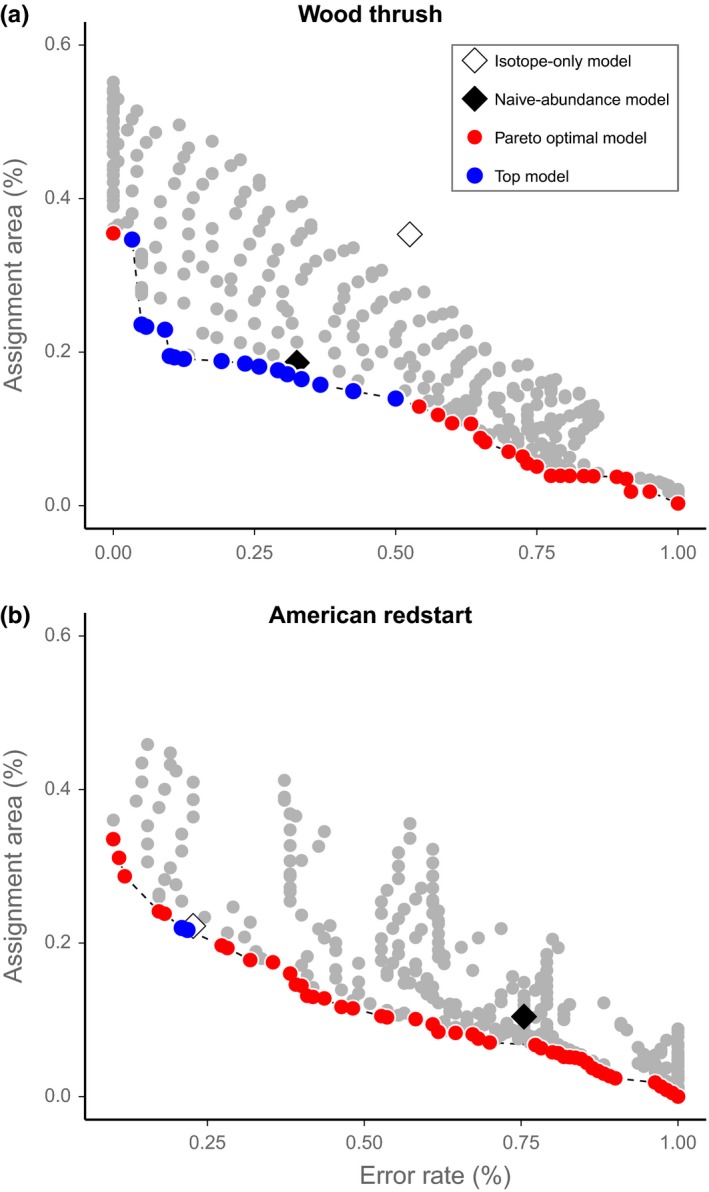
Assignment area and error rate of the 442 (a) Wood Thrush and (b) American Redstart assignment models

For both Wood Thrush and American Redstart, the majority of the 442 assignment models performed poorly relative to the best models, indicating only a small range of weightings provided reasonable solutions for incorporating abundance into assignments (Figure 3). The isotope‐only models had moderate rates for both species (Wood Thrush: 52%; American Redstart: 23%) but also relatively large assignment areas (Figures 1b and 2b; Wood Thrush: 35%; American Redstart: 22%; Table 2). In contrast, the naive‐abundance models had low assignment areas (Figures 1c and 2c; Wood Thrush: 19%; American Redstart: 10%) but high error rates (Wood Thrush: 32%; American Redstart: 75%; Table 2).

**Table 2:** Comparison of the assignment performance of the isotope‐only models, naive‐abundance models, and top abundance models for six species of Neotropical migratory birds. For each model, assignment area is the mean proportion of cells classified as “likely” origins across all individuals and error rate is the proportion of individuals whose actual breeding origin was incorrectly classified as an “unlikely” origin. See text for details on selecting the top model for each species

Species	Isotope‐only model		Naïve‐abundance model		Top abundance model	
	Area (%)	Error (%)	Area (%)	Error (%)	Area (%)	Error (%)
Wood Thrush	35	53	19	33	23	6
American Redstart	22	23	10	75	22	21
Ovenbird	25	63	14	53	18	33
Northern Parula	35	37	18	30	23	20
Black‐and‐White Warbler	25	53	16	47	24	21
Prairie Warbler	41	56	22	93	39	56

The use of Pareto optimality provided an efficient means of eliminating poorly performing models. For Wood Thrush, 405 models (92%) were not Pareto optimal, leaving 37 models along the frontier (Figure 3). Only 16 of the 37 Pareto optimal models (43%) outperformed the isotope‐only model with regard to both assignment area and error rate (Figure 3). In general, the best performing Wood Thrush models tended to weight the isotope data by a power <1 (Table S7), indicating that assignments performed best when the likelihoods, but not the prior, were slightly flattened compared to their original distribution.

**Figure 4:**
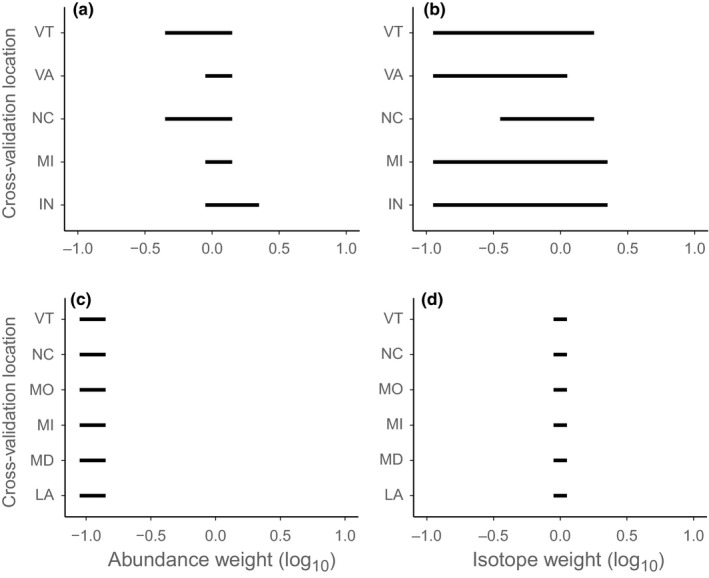
Sampling site cross‐validation results for Wood Thrush (a‐b) and American Redstart (c‐d). For each site shown on the *y*‐axis, the bars show the range of abundance weights (a, c) and isotope weights (b, d) from the top models when all individuals from that site are removed from the analysis. Top models were determined by comparing the assignment area and error rate of the Pareto optimal models and the assignment area and error rate of the isotope‐only models

The sampling location validation procedure revealed that the Wood Thrush isotope weights were sensitive to the inclusion of the North Carolina site (Figure 4). Previous analysis of these data indicated that the isotope data performed poorly for these individuals, with only 40% (13/32) of the individuals correctly assigned to their breeding site (Rushing et al., 2014). Downweighting the isotope data likely improved the assignment of these individuals by flattening the likelihood distribution relative to the prior, thus giving more weight to locations with higher abundance (and in this case, the true origin). As a result, including the North Carolina samples in the current analysis favored models that had lower isotope weights than when these individuals were not included (Figure 4). Aside from the influence of the North Carolina site, the cross‐validation procedure indicated that the top Wood Thrush models were not highly sensitive to the sampling locations included in the analysis.

For American Redstart, 395 models (90%) were not Pareto optimal, leaving 47 models along the frontier (Figure 3). In contrast to the Wood Thrush models, the isotope‐only model was very close to the Pareto frontier and as a result, only two of the 46 Pareto optimal abundance models outperformed the isotope‐only model with regard to both assignment area and error rate (Figure 3). Both models heavily downweighted abundance (weight range = 10^−1.0^–10^−0.9^) and but did not weight the isotope data (Table S8). The isotope weights and the abundance weights were similar across all cross‐validation models for American Redstart (Figure 4), indicating that the top Redstart models were unaffected by sample site location.

In general, the multispecies validation indicated that the top models for Wood Thrush and American Redstart performed better than the isotope‐only model for the four other species included in our analysis (Table 2). For Ovenbird, Black‐and‐White Warbler, and Northern Parula, 15 of the 16 of the top Wood Thrush models outperformed the respective isotope‐only models with regard to both assignment area and error rate (Tables S9–S11). In contrast, the top American Redstart models performed poorly for these species. For Prairie Warbler, none of the top Wood Thrush models outperformed the isotope‐only model (Table S12), although the top American Redstart models did provide slight improvements in assignment area with no increase in error rate.

As for Wood Thrush and American Redstart, the naive‐abundance models for the remaining four species performed poorly, with a mean error rate of 56% (range = 30%–93%). Thus, although the naive‐abundance models greatly reduced the assignment area relative to the isotope‐only models (mean decrease in assignment area compared to isotope‐only models = 46%), they performed poorly compared to both the isotope‐only model and the top weighted abundance models for all species (Table 2).

The divergent results for Wood Thrush, Ovenbird, Black‐and‐White Warbler, and Northern Parula, on the one hand, and American Redstart and Prairie Warbler, on the other hand, suggest that the performance of abundance models is conditional on the underlying distribution of abundance. To test this hypothesis, we fit a negative exponential distribution to the abundance data for each species and compared the rate parameter across species. Patchy abundance distributions are expected to be characterized by steeply declining distributions and large rate parameters. More even abundance distributions should be less steep and have lower rate parameters. Consistent with our hypothesis, the rate parameters for American Redstart (23.3, 95% CI = 22.8‐23.9; Figure S2) and Prairie Warbler (19.2, 18.3‐20.1) are much larger than the parameters for Wood Thrush (8.4, 8.2‐8.7), Ovenbird (8.2, 8.0‐8.4), Northern Parula (12.3, 11.9‐12.7), and Black‐and‐White Warbler (8.1, 7.8‐8.3).

## 4 Discussion

Given the low resolution of many intrinsic markers, including abundance in assignment models is appealing because it can often greatly increase the precision of assignments. However, in all assignment models that use intrinsic markers, there is an inherent trade‐off between assignment error and precision. Using known‐origin stable‐hydrogen isotope samples of six Neotropical migratory bird species, we show that the increased precision of models that include abundance is often offset by a large increase in assignment error. We demonstrate that proper weighting of the abundance and isotope data can result in models with higher precision and, in some cases, lower error than models based on isotopes alone. These results confirm that breeding abundance can be an important source of information for studies investigating large‐scale movements of migratory birds and potentially other taxa.

Although our assignment models involved known‐origin birds, their chief application will be estimating migratory connectivity to breeding areas for adult birds captured in the stationary nonbreeding period or while on migration. We emphasize that combining breeding abundance and stable isotopes in assignment models will yield valid estimates of migratory connectivity for adults but not for immature birds because breeding abundance can be a poor indicator of regional productivity (Van Horne, 1983). Studies aimed at determining the natal origins of immature birds could instead estimate range‐wide variation in fecundity with data from the Monitoring Avian Productivity and Survival (MAPS) program (Desante, Burton, Saracco, & Walker, 1995).

The use of Pareto optimality allowed us to define explicitly the trade‐off between precision and error and in that way focus only on models that provided potential solutions to the proper weighting of abundance and isotope data. For the four species with relatively even abundance distributions (Wood Thrush, Ovenbird, Black‐and‐White Warbler, and Northern Parula), several models along the Pareto frontier had both lower error and lower assignment area than models based on isotopes alone. Across these four species, models that weighted the isotope data by 10^−0.6^–10^−0.8^ but left the abundance data unweighted provided the best performance. Further downweighting the isotope data resulted in a large increase in error rate for Northern Parula and Black‐and‐White Warbler, offsetting the improved assignment area for the other species.

In contrast, including abundance did not provide an unequivocal improvement in assignment performance for the remaining two species (American Redstart and Prairie Warbler). For these species, the isotope‐only model was close to the Pareto frontier. In general, this means that although including abundance in the model will decrease assignment area, it will also increase assignment error. Nevertheless, weighting abundance by 10^−1^ but leaving the isotope data unweighted did provide a slight decrease in assignment area without substantial increase in error rate for these species. Therefore, this combination appears to provide a reasonable solution for these two species.

These results indicate that the distribution of breeding abundance plays a critical role in the performance of assignment models. For species with breeding ranges that are characterized by large areas of very low abundance and a few small centers of high abundance, this patchy distribution likely magnifies the influence of abundance data in the assignment model, overwhelming the information provided by the isotope data and leading to high posterior probabilities for only the high abundance sites (Hobson et al., 2014). As a result, even models that heavily downweight the abundance data perform relatively poorly compared to the isotope‐only model. For species with relatively even distributions, in contrast, downweighting of the abundance data was unnecessary to give appropriate weight to the isotope data. Thus, we suggest that including abundance in assignment models has the most potential to improve assignment of species with relatively less patchy and more even distribution of breeding abundance.

One of the primary assumptions, often made implicitly, behind adding abundance to assignment models is that at any given winter site, individuals mix in frequencies relative to their breeding abundance (González‐Prieto et al., 2011). In other words, the logic behind this approach assumes that there is no migratory connectivity. However, several decades of research on migratory connectivity have shown that complete mixing is the exception rather than the rule. Most migratory species show some degree of migratory connectivity, with different breeding populations migrating to different winter locations. Because the abundance model ignores this nonrandom mixing of individuals, the model will tend to obscure true patterns of migratory connectivity by biasing all assignments toward high abundance locations. Thus, we caution researchers to carefully consider adding abundance to assignment models when they know or expect some degree of migratory connectivity.

Of course, for species for which there is no a priori information about migratory connectivity, it will be difficult to determine whether or how to include abundance in assignment models. When known‐origin samples are available, researchers should always test model performance before assignment of unknown‐origin individuals. In cases where known‐origin samples are not available, we suggest researchers consider the following recommendations:

1. Do not use unweighted abundance data: For all six species included in our analysis, the naive‐abundance model was not an optimal solution to model weighting and some cases provided unacceptably high error rates. Therefore, researchers should not use the unweighted abundance estimates in assignment models.
2. Downweight abundance for species with patchy distributions: Our results for American Redstart and Prairie Warbler, two species characterized by very patchy breeding distributions (exponential rate parameters > 19), suggest that including abundance in assignments provides only a small improvement over the isotope‐only model and only when the abundance data were heavily downweighted. Therefore, we recommend that researchers first characterize the patchiness of breeding distributions using the exponential curve‐fitting approach described above. For species with patchy distributions (rate parameter > ~15–20), abundance data should only be included if it is heavily downweighted (10^−1^–10^−0.9^).
3. Moderately downweight isotope data for species with even abundance: For species with more even distributions of breeding abundance (rate parameter < 12), the best models tended to moderately downweight the isotope but not the abundance data. Therefore, we recommend for species with rate parameters < 12, isotope data should be weighted 10^−0.6^–10^−0.8^ and abundance data should be unweighted.

Even when researchers follow these guidelines, we recommend comparing the results of the abundance model to the results of assignments based on stable isotopes only. As we show here, the models along the Pareto frontier define the upper bound of assignment performance using abundance and stable‐hydrogen isotopes and, in some cases, the performance of abundance models can be poor compared to the isotope‐only model. As a result, large discrepancies between the two models should be investigated, reported, and carefully interpreted. Ultimately, however, including abundance in assignment models should be viewed as a preliminary step to improving estimates of migratory connectivity for any species. Occupancy probabilities calculated from species distribution models offer a potentially promising alternative to abundance (Fournier et al., 2016); in part, this approach could lessen the influence of areas with particularly high or low abundance. However, the largest improvements in assignments likely will come from incorporating multiple intrinsic markers (e.g., DNA, morphology, or other isotopes), each of which provides complimentary information about the origin of an individual. Given the low cost and large sample sizes associated with assaying intrinsic markers, assignment models based on these methods will continue to provide important insights into the migratory movements of many species.

## Data Archiving

Upon acceptance, the data used in this manuscript, along with all necessary code to reproduce the analyses, will be deposited permanently in the Smithsonian Digital Data Repository. Once archived, the data and code will be freely available to users.

## Conflict of Interest

None declared.
