# Supplementary material for: Incorporating breeding abundance into spatial assignments on continuous surfaces
Source: Ecol Evol. 2017 Apr 21;7(11):3847–55. doi: 10.1002/ece3.2605 (PMC5468143; doi:10.1002/ece3.2605)
Supplement: Supplementary file 1 [file ECE3-7-3847-s001.docx]

# Appendix A

### Details of species' sampling

Table S1: Wood Thrush sampling sites with geographic coordinates and the number of individuals sampled.

| Model | Latitude | Longitude | n |
| --- | --- | --- | --- |
| North Carolina | 35.40 | -83.12 | 32 |
| Virginia | 38.71 | -77.15 | 27 |
| Indiana | 38.84 | -86.82 | 28 |
| Michigan | 42.16 | -85.47 | 6 |
| Vermont | 44.51 | -73.15 | 27 |

Table S2: American Redstart sampling sites with geographic coordinates and the number of individuals sampled.

| Model | Latitude | Longitude | n |
| --- | --- | --- | --- |
| Louisiana | 30.30 | -89.67 | 20 |
| North Carolina | 35.40 | -83.12 | 14 |
| Missouri | 38.35 | -93.55 | 20 |
| Maryland | 38.54 | -76.75 | 20 |
| Vermont | 44.54 | -73.10 | 16 |
| Michigan | 44.65 | -84.13 | 20 |

Table S3: Ovenbird sampling sites with geographic coordinates and the number of individuals sampled.

| Model | Latitude | Longitude | n |
| --- | --- | --- | --- |
| North Carolina | 35.40 | -83.12 | 5 |
| Missouri | 38.35 | -93.55 | 5 |
| Maryland | 38.54 | -76.75 | 5 |
| West Virginia | 38.45 | -79.27 | 5 |
| Vermont | 44.54 | -73.10 | 5 |
| Michigan | 44.65 | -84.13 | 5 |

Table S4: Northern Parula sampling sites with geographic coordinates and the number of individuals sampled.

| Model | Latitude | Longitude | n |
| --- | --- | --- | --- |
| Louisiana | 30.30 | -89.67 | 5 |
| Georgia | 31.88 | -81.61 | 5 |
| North Carolina | 35.40 | -83.12 | 5 |
| Missouri | 38.35 | -93.55 | 5 |
| Maryland | 38.54 | -76.75 | 2 |
| Maine | 44.86 | -68.65 | 5 |

Table S5: Black-and-White Warbler sampling sites with geographic coordinates and the number of individuals sampled from each age/sex class

| Model | Latitude | Longitude | n |
| --- | --- | --- | --- |
| North Carolina | 35.40 | -83.12 | 10 |
| Maryland | 38.54 | -76.75 | 3 |
| Vermont | 44.54 | -73.20 | 2 |
| Maine | 44.86 | -68.65 | 5 |

.

Table S6: Prairie Warbler sampling sites with geographic coordinates and the number of individuals sampled from each age/sex class.

| Model | Latitude | Longitude | n |
| --- | --- | --- | --- |
| Louisiana | 31.10 | -93.22 | 5 |
| Georgia | 31.88 | -81.61 | 5 |
| North Carolina | 35.22 | -82.43 | 5 |
| Virginia | 38.71 | -77.15 | 4 |
| Missouri | 38.35 | -93.55 | 2 |
| Maryland | 38.54 | -76.75 | 1 |
| New York | 42.75 | -78.84 | 5 |

### Assignment model performance

Table S7: Model performance for the assignment of Wood Thrush using the isotope-only, naive-abundance, and top Wood Thrush and American Redstart models.

| Model | Abundance Weight | Isotope Weight | Area (%) | Error (%) |
| --- | --- | --- | --- | --- |
| Isotope-only | NA | 0.0 | 35.34 | 52.50 |
| Naive-abundance | 0.0 | 0.0 | 18.61 | 32.50 |
| Wood Thrush | -0.3 | -0.6 | 34.67 | 3.33 |
| Wood Thrush | 0.0 | -0.8 | 23.59 | 5.00 |
| Wood Thrush | 0.0 | -0.7 | 23.30 | 5.83 |
| Wood Thrush | 0.0 | -0.6 | 22.93 | 9.17 |
| Wood Thrush | 0.1 | -0.9 | 19.48 | 10.00 |
| Wood Thrush | 0.1 | -0.8 | 19.32 | 10.83 |
| Wood Thrush | 0.1 | -0.7 | 19.11 | 12.50 |
| Wood Thrush | 0.1 | -0.6 | 18.84 | 19.17 |
| Wood Thrush | 0.1 | -0.5 | 18.51 | 23.33 |
| Wood Thrush | 0.1 | -0.4 | 18.11 | 25.83 |
| Wood Thrush | 0.1 | -0.3 | 17.64 | 29.17 |
| Wood Thrush | 0.1 | -0.2 | 17.11 | 30.83 |
| Wood Thrush | 0.1 | -0.1 | 16.48 | 33.33 |
| Wood Thrush | 0.1 | 0.0 | 15.75 | 36.67 |
| Wood Thrush | 0.1 | 0.1 | 14.91 | 42.50 |
| Wood Thrush | 0.1 | 0.2 | 13.95 | 50.00 |
| American Redstart | -1.0 | 0.0 | 33.59 | 42.50 |
| American Redstart | -0.9 | 0.0 | 33.20 | 40.83 |

Table S8: Model performance for the assignment of American Redstart using the isotope-only, naive-abundance, and top Wood Thrush and American Redstart models.

| Model | Abundance Weight | Isotope Weight | Area (%) | Error (%) |
| --- | --- | --- | --- | --- |
| Isotope-only | NA | 0.0 | 22.23 | 22.73 |
| Naive-abundance | 0.0 | 0.0 | 10.41 | 75.45 |
| Wood Thrush | -0.3 | -0.6 | 22.92 | 61.82 |
| Wood Thrush | 0.0 | -0.8 | 16.44 | 79.09 |
| Wood Thrush | 0.0 | -0.7 | 15.46 | 78.18 |
| Wood Thrush | 0.0 | -0.6 | 14.59 | 78.18 |
| Wood Thrush | 0.1 | -0.9 | 14.40 | 79.09 |
| Wood Thrush | 0.1 | -0.8 | 13.53 | 79.09 |
| Wood Thrush | 0.1 | -0.7 | 12.70 | 79.09 |
| Wood Thrush | 0.1 | -0.6 | 11.99 | 79.09 |
| Wood Thrush | 0.1 | -0.5 | 11.37 | 78.18 |
| Wood Thrush | 0.1 | -0.4 | 10.83 | 78.18 |
| Wood Thrush | 0.1 | -0.3 | 10.34 | 77.27 |
| Wood Thrush | 0.1 | -0.2 | 9.82 | 75.45 |
| Wood Thrush | 0.1 | -0.1 | 9.25 | 75.45 |
| Wood Thrush | 0.1 | 0.0 | 8.60 | 75.45 |
| Wood Thrush | 0.1 | 0.1 | 7.89 | 76.36 |
| Wood Thrush | 0.1 | 0.2 | 7.16 | 79.09 |
| American Redstart | -1.0 | 0.0 | 21.97 | 20.91 |
| American Redstart | -0.9 | 0.0 | 21.72 | 21.82 |

Table S9: Model performance for the assignment of Ovenbird using the isotope-only, naive-abundance, and top Wood Thrush and American Redstart models.

| Model | Abundance Weight | Isotope Weight | Area (%) | Error (%) |
| --- | --- | --- | --- | --- |
| Isotope-only | NA | 0.0 | 24.59 | 63.33 |
| Naive-abundance | 0.0 | 0.0 | 13.63 | 53.33 |
| Wood Thrush | -0.3 | -0.6 | 25.41 | 16.67 |
| Wood Thrush | 0.0 | -0.8 | 19.06 | 33.33 |
| Wood Thrush | 0.0 | -0.7 | 18.46 | 33.33 |
| Wood Thrush | 0.0 | -0.6 | 17.86 | 33.33 |
| Wood Thrush | 0.1 | -0.9 | 16.58 | 50.00 |
| Wood Thrush | 0.1 | -0.8 | 16.09 | 50.00 |
| Wood Thrush | 0.1 | -0.7 | 15.61 | 50.00 |
| Wood Thrush | 0.1 | -0.6 | 15.17 | 53.33 |
| Wood Thrush | 0.1 | -0.5 | 14.72 | 50.00 |
| Wood Thrush | 0.1 | -0.4 | 14.27 | 50.00 |
| Wood Thrush | 0.1 | -0.3 | 13.80 | 50.00 |
| Wood Thrush | 0.1 | -0.2 | 13.29 | 56.67 |
| Wood Thrush | 0.1 | -0.1 | 12.70 | 66.67 |
| Wood Thrush | 0.1 | 0.0 | 11.97 | 66.67 |
| Wood Thrush | 0.1 | 0.1 | 11.04 | 66.67 |
| Wood Thrush | 0.1 | 0.2 | 9.95 | 63.33 |
| American Redstart | -1.0 | 0.0 | 24.60 | 50.00 |
| American Redstart | -0.9 | 0.0 | 24.28 | 50.00 |

Table S10: Model performance for the assignment of Northern Parula using the isotope-only naive-abundance, and top Wood Thrush and American Redstart models.

| Model | Abundance Weight | Isotope Weight | Area (%) | Error (%) |
| --- | --- | --- | --- | --- |
| Isotope-only | NA | 0.0 | 35.21 | 36.67 |
| Naive-abundance | 0.0 | 0.0 | 17.91 | 30.00 |
| Wood Thrush | -0.3 | -0.6 | 37.82 | 3.33 |
| Wood Thrush | 0.0 | -0.8 | 23.71 | 20.00 |
| Wood Thrush | 0.0 | -0.7 | 23.33 | 20.00 |
| Wood Thrush | 0.0 | -0.6 | 22.78 | 20.00 |
| Wood Thrush | 0.1 | -0.9 | 17.90 | 20.00 |
| Wood Thrush | 0.1 | -0.8 | 17.74 | 20.00 |
| Wood Thrush | 0.1 | -0.7 | 17.49 | 20.00 |
| Wood Thrush | 0.1 | -0.6 | 17.13 | 20.00 |
| Wood Thrush | 0.1 | -0.5 | 16.72 | 20.00 |
| Wood Thrush | 0.1 | -0.4 | 16.23 | 23.33 |
| Wood Thrush | 0.1 | -0.3 | 15.73 | 23.33 |
| Wood Thrush | 0.1 | -0.2 | 15.18 | 23.33 |
| Wood Thrush | 0.1 | -0.1 | 14.59 | 23.33 |
| Wood Thrush | 0.1 | 0.0 | 13.96 | 30.00 |
| Wood Thrush | 0.1 | 0.1 | 13.33 | 36.67 |
| Wood Thrush | 0.1 | 0.2 | 12.62 | 36.67 |
| American Redstart | -1.0 | 0.0 | 34.86 | 30.00 |
| American Redstart | -0.9 | 0.0 | 34.63 | 26.67 |

Table S11: Model performance for the assignment of Black-and-White Warbler using the isotope-only, naive-abundance, and top Wood Thrush and American Redstart models.

| Model | Abundance Weight | Isotope Weight | Area (%) | Error (%) |
| --- | --- | --- | --- | --- |
| Isotope-only | NA | 0.0 | 25.28 | 52.63 |
| Naive-abundance | 0.0 | 0.0 | 16.09 | 47.37 |
| Wood Thrush | -0.3 | -0.6 | 34.77 | 0.00 |
| Wood Thrush | 0.0 | -0.8 | 24.73 | 21.05 |
| Wood Thrush | 0.0 | -0.7 | 24.35 | 21.05 |
| Wood Thrush | 0.0 | -0.6 | 23.97 | 21.05 |
| Wood Thrush | 0.1 | -0.9 | 20.31 | 21.05 |
| Wood Thrush | 0.1 | -0.8 | 20.13 | 21.05 |
| Wood Thrush | 0.1 | -0.7 | 19.92 | 21.05 |
| Wood Thrush | 0.1 | -0.6 | 19.62 | 21.05 |
| Wood Thrush | 0.1 | -0.5 | 19.22 | 21.05 |
| Wood Thrush | 0.1 | -0.4 | 18.61 | 21.05 |
| Wood Thrush | 0.1 | -0.3 | 17.70 | 21.05 |
| Wood Thrush | 0.1 | -0.2 | 16.44 | 21.05 |
| Wood Thrush | 0.1 | -0.1 | 14.90 | 26.32 |
| Wood Thrush | 0.1 | 0.0 | 13.31 | 47.37 |
| Wood Thrush | 0.1 | 0.1 | 11.78 | 47.37 |
| Wood Thrush | 0.1 | 0.2 | 10.37 | 52.63 |
| American Redstart | -1.0 | 0.0 | 25.13 | 47.37 |
| American Redstart | -0.9 | 0.0 | 25.07 | 47.37 |

Table S12: Model performance for the assignment of Prairie Warbler using the isotope-only, naive-abundance, and top Wood Thrush and American Redstart models.

| Model | Abundance Weight | Isotope Weight | Area (%) | Error (%) |
| --- | --- | --- | --- | --- |
| Isotope-only | NA | 0.0 | 40.55 | 55.56 |
| Naive-abundance | 0.0 | 0.0 | 22.12 | 92.59 |
| Wood Thrush | -0.3 | -0.6 | 40.48 | 81.48 |
| Wood Thrush | 0.0 | -0.8 | 26.09 | 85.19 |
| Wood Thrush | 0.0 | -0.7 | 25.92 | 85.19 |
| Wood Thrush | 0.0 | -0.6 | 25.65 | 85.19 |
| Wood Thrush | 0.1 | -0.9 | 20.51 | 88.89 |
| Wood Thrush | 0.1 | -0.8 | 20.44 | 92.59 |
| Wood Thrush | 0.1 | -0.7 | 20.30 | 92.59 |
| Wood Thrush | 0.1 | -0.6 | 20.12 | 92.59 |
| Wood Thrush | 0.1 | -0.5 | 19.89 | 92.59 |
| Wood Thrush | 0.1 | -0.4 | 19.63 | 92.59 |
| Wood Thrush | 0.1 | -0.3 | 19.31 | 96.30 |
| Wood Thrush | 0.1 | -0.2 | 18.94 | 96.30 |
| Wood Thrush | 0.1 | -0.1 | 18.46 | 96.30 |
| Wood Thrush | 0.1 | 0.0 | 17.82 | 96.30 |
| Wood Thrush | 0.1 | 0.1 | 16.92 | 100.00 |
| Wood Thrush | 0.1 | 0.2 | 15.95 | 100.00 |
| American Redstart | -1.0 | 0.0 | 39.22 | 55.56 |
| American Redstart | -0.9 | 0.0 | 38.97 | 55.56 |

### Abundance distributions

Figure S1: Relative breeding abundances and sampling locations of Ovenbirds, Northern Parula, Black-and-White Warblers, and Prairie Warblers

Figure S2: Distribution of relative breeding abundances and fitted negative exponential distributions for each of the six species
