# Supplementary material for: Incorporating breeding abundance into spatial assignments on continuous surfaces
Source: Ecol Evol. 2017 Apr 21;7(11):3847–55. doi: 10.1002/ece3.2605 (PMC5468143; doi:10.1002/ece3.2605)
Supplement: Supplementary file 2 [file ECE3-7-3847-s002.pdf]

Supplementary information:

Annotated code for assignment models and Pareto optimality

Clark S. Rushing, Peter P. Marra, & Colin E. Studds

October 3, 2016

The following code provides a step-by-step guide to performing the analysis presented in our manuscript. We have done out best to clearly annotate each step so that the reviewers can fully interpret our methods and our results. Once accepted, we will include a modified version of this document as a supplement so that readers will have access to our data and code.

The code used to estimate cumulative probability thresholds was adapted from code kindly provided by the reviewer. We note the although our code includes the smoothing spline approach recommended by the reviewer, the remainder of our method differs from that of S.L. Van Wilgenberg et al. (*unpub*) in that we treat the isoscape basemaps as dataframes rather than raster objects. In our experience, this choice makes manipulation of the basemap objects and visualizing/saving model output more intuitive. However, the results from either approach are identical so this is purely a matter of preference.

The code requires several packages:

```
require(dplyr)
require(tidyr)
require(purrr)
require(ggplot2)

options(width = 60)
```

Isotope-based assignment

To assign individuals using the $\delta^2 H_f$ data, our assignment requires a vector **D** containing the $\delta^2 H_f$ values for each of the n individuals and a vector **B** containing the predicted $\delta^2 H_f$ values for each of the b breeding cells. To estimate the likelihood that each breeding cell b is the origin of each individual, we first create an empty matrix with dimensions $b \times n$ that will store the likelihood values:

```
  iso_like <- matrix(ncol = length(D), nrow = length(B)) # One row/cell; One column/indv

  colnames(iso_like) <- paste("Indv_", seq(1:length(dd)), sep = "")
```

For reasons that are not entirely clear to us, assigning column names to the matrix greatly speeds up the assignment.

Next, we loop over each individual and use the `dnorm` function to estimate the likelihood that each cell is the origin for that individual:

```
    for(i in 1:length(D)){ # For each individual
        iso_like[, i] <- dnorm(D[i], mean = B, sd = 12)
    }
```

We assume a standard deviation = 12 based on within-site variation of $\delta^2 H_f$ values for our known-origin birds. Next, we use a series of functions that convert the likelihoods into binary (i.e., likely vs. unlikely) origin classifications:

```r
  iso_data <- as.data.frame(iso_like) %>%
      ## Covert likelihood matrix into 'long' format
      gather(indv, iso_like) %>%
      ## Perform following calculations per individual
      group_by(indv) %>%
      ## Convert the likelihood surface into probability surface and
      ## add columns w/ probabilities, latitude, and longitude
      mutate(iso_prob = iso_like / sum(iso_like),
             lat = basemap$lat, lon = basemap$lon) %>%
      ungroup() %>%
      ## Create nested data.frame to store cumulative probability estimates for each indv
      nest(-indv) %>%
      ## Using smoothing spline to predict prob. threshold that includes 67% of cum. prob.
      ## Store threshold as iso_cut
      mutate(fit = map(data, ~ predict(smooth.spline(cumsum(sort(.$iso_prob)),
                                       sort(.$iso_prob), spar = 0.1), 0.33)),
             iso_cut = map_dbl(fit, "y")) %>%
      ## Remove smoothing spline results & unnest
      select(-fit) %>%
      unnest() %>%
      ## Classify cells as likely if prob. > threshold; add as new column
      mutate(iso_origin = ifelse(iso_prob > iso_cut, 1, 0)) %>%
      ## Select columns to be saved in final output
      select(indv, lat, lon, iso_cut, iso_like, iso_prob, iso_origin)
```

The heart of this function is estimating the probability threshold that includes 67% of the cumulative probability of origin. To accomplish this, the data frame that contains the isotope-based probabilities is *nested* by individual. In essence, this approach allows us to fit a model separately for each individual and store the model output in our original data frame. After nesting the data frame by individual, we use the `map` function to add a new column called *fit* which contains the output from the `smooth.spline` function. As outlined by Van Wilgenburg et al. (unpub.), this function uses the isotope-based probabilities to estimate the probability threshold that accounts for (in this case) 67% of the cumulative probability of origin. Next, we create a new column *iso_cut* which contains, for each individual, the probability threshold for each individual. Finally, we remove the *fit* column, `unnest` the data frame, and the add a new column *iso_origin* which has a value of 1 (likely) if the cell probability is > *iso_cut* and 0 otherwise. The final object *iso_data* is a data frame containing the individual IDs, latitude and longitude of each breeding cell, the probability threshold, the cell likelihood values, the cell probabilities, and the likely/unlikely classifications.

To aid application of this code to multiple species or sites, we combine all of the above code into a function called `iso_assign`:

```r
iso_assign <- function(D, B, lat, lon, odds) {
  #### Create empty matrix to store likelihoods
  iso_like <- matrix(ncol = length(D), nrow = length(B)) # One row/cell; One column/indv

  colnames(iso_like) <- paste("Indv_", seq(1:length(D)), sep = "")

  #### Estimate likelihoods
    for(i in 1:length(D)){ # For each individual
        iso_like[, i] <- dnorm(D[i], mean = B, sd = 12)
    }


  #### Create output object
```

```r
  iso_data <- as.data.frame(iso_like) %>%       # Convert likelihood matrix to data frame
      gather(indv, iso_like) %>%
      group_by(indv) %>%
      mutate(iso_prob = iso_like / sum(iso_like),
             lat = lat, lon = lon) %>%
      ungroup() %>%
      nest(-indv) %>%
      mutate(fit = map(data, ~ predict(smooth.spline(cumsum(sort(.$iso_prob)),
                                  sort(.$iso_prob), spar = 0.1), 1 - odds)),
             iso_cut = map_dbl(fit, "y")) %>%
      select(-fit) %>%
      unnest() %>%
      mutate(iso_origin = ifelse(iso_prob > iso_cut, 1, 0)) %>%
      select(indv, lat, lon, iso_cut, iso_like, iso_prob, iso_origin)

  return(iso_data)
}
```

The function takes a vector **D** containing the sampled δ^2H_f values, a vector **B** containing the predicted δ^2H_f for each breeding cell, vectors *lat* and *lon* containing the latitude and longitude of each breeding cell, and the chosen *odds*.

Model performance

We use two metrics to measure the performance of our assignment models. First, we determine the proportion of individuals whose **true** origin cell is *incorrectly* classified as unlikely, which we term the assignment error rate. A good assignment model should minimize error rate. Assuming we know the true breeding origin cell for each individual, we can estimate the error rate using the following function:

```r
error_rate <- function(df, origin_cell){
    # Convert vector of likely/unlikely classifications to cell x indv matrix
      origin <- matrix(df$iso_origin, ncol = length(unique(df$indv)), byrow = FALSE)
    # Extract classification for the true origin of each individual
      correct <- origin[cbind(origin_cell, seq_along(origin_cell))]
    # Estimate error rate
      err_rate <- 1 - mean(correct)
  return(err_rate)
}

iso_error <- error_rate(df = iso_data, origin_cell = origin_cell)
```

Here, `origin_cell` is a numeric vector of length n indicating which cell in **B** is the true origin of each individual. The function then extracts the classification of each individual's true origin (1 = likely, 0 = unlikely), and estimates the proportion which are incorrectly classified as unlikely.

Second, we estimate the mean proportion of the breeding cells that are classified as likely origins, which we term *assignment area*. Ideally, a good assignment model should minimize assignment area. The following function estimates the mean area from the isotope-based assignment:

```r
area_assign <- function(df){
  #### Average # cells identified as likely for isotope only assignment
    area <- df %>%
```

```
    group_by(indv) %>%
    summarise(cells = sum(iso_origin), area = cells / length(iso_origin)) %>%
    .$area
  return(mean(area))
}

iso_area <- area_assign(df = iso_data)
```

Incorporating abundance

Next, we incorporate the relative abundance into the assignment using Bayes rule:

$$f(b_i|y*) = \frac{f(y*|b_i)f(b_i)}{\sum_{i=1}^{B} f(y*|b_i)f(b_i)}$$

where $f(b_i|y*)$ is the posterior probability that an individual with $\delta^2 H_f = y*$ originated from cell i, $f(y*|b_i)$ is the likelihood of assignment to breeding cell i, and $f(b_i)$ is the relative abundance (i.e., the prior probability) of cell i. Because we already estimated the likelihoods in the steps above, estimating the posterior probability for each cell is simply a matter of multiplying the likelihoods by the relative abundances and then relativizing this product to sum to 1:

```
post_prob <- iso_data %>%
             ## Perform estimation on each individual separately
             group_by(indv) %>%
             ## Estimate posterior probability for each cell, using rel. abun. as prior
              mutate(rel_prob = iso_like * A,
                     post_prob = rel_prob / sum(rel_prob))
```

where **A** is a vector containing the relative abundance values for each of the b breeding cells (note that for **A** to be a proper prior, it must sum to 1). To convert the `post_prob` estimates into likely/unlikely breeding origins, we would then follow the same steps as above for the isotope model. We could likewise use the `error_rate` and `area_assign` functions to measure model performance.

Finding optimal weights

The primary purpose of this analysis is to determine whether weighting the likelihood and prior information can improve the model performance compared to the isotope-only model. To do this, we raise the likelihood and prior values to powers from 10^{-1} to 10 and measure model performance using the `error_rate` and `area_assign` functions. Weights < 1 flatten the likelihood/prior distributions (giving relatively more weight to smaller values) and weights > 1 sharpen the distributions (giving relatively less weight to smaller values). . First, we create a data frame containing the 441 weighting combinations:

```
  weight_range <- seq(from = -1, to = 1, by = .1)
  weights <- expand.grid(x = 10 ^ weight_range, y = 10 ^ weight_range)
  names(weights) <- c("iso_weight", "abun_weight")
```

Assignment results are stored in as a list, with one element for each of the 441 models. To conduct the analysis, we next create an empty list, loop over each weight combination, estimate the posterior probabilities for each individual, and then covert the posterior probabilities into likely/unlikely origins:

```r
  wght_summ <- list()

  # Estimate posteriors under each weighting combination
  for(i in 1:nrow(weights)){ # For each weighting combination
    wght_summ[[i]] <- iso_data %>%
      group_by(indv) %>%
      mutate(rel_prob = A ^ weights$abun_weight[i] * iso_like ^ weights$iso_weight[i],
             post_prob = rel_prob / sum(rel_prob)) %>%
      ungroup() %>%
      select(-rel_prob) %>%
      ## Nest by individual
      nest(-indv) %>%
      ## Estimate cumulative probability and estimate cutoff value for each individual
      mutate(fit = purrr::map(data, ~ predict(smooth.spline(cumsum(sort(.$post_prob)),
                                               sort(.$post_prob), spar = 0.1), 0.33)),
             wght_cutoff = map_dbl(fit, "y")) %>%
      ## Remove spline predictions & unnest dataframe
      select(-fit) %>%
      unnest() %>%
      ## Reclassify cells as likely/unlikely based on cumulative prob
      mutate(wght_origin = ifelse(post_prob > wght_cutoff, 1, 0))
  }
```

where `iso_data` is the output from the `iso_assign` function. As for the isotope assignment code, we package the above code in a custom function to aid portability and flexibility:

```r
# Weighted Models
weight_assign <- function(iso_assign, A, min_weight, max_weight, odds){

  # Create data frame containing all weight combinations
  weight_range <- seq(from = min_weight, to = max_weight, by = .1)
  weights <- expand.grid(x = 10 ^ weight_range, y = 10 ^ weight_range)
  names(weights) <- c("iso_weight", "abun_weight")

  wght_summ <- list()

  # Estimate posteriors under each weighting combination
  for(i in 1:nrow(weights)){ # For each weighting combination
    wght_summ[[i]] <- iso_assign %>%
      group_by(indv) %>%
      mutate(temp_prob = A^weights$abun_weight[i] * iso_like^weights$iso_weight[i],
             wght_prob = temp_prob / sum(temp_prob)) %>%
      ungroup() %>%
      select(-temp_prob) %>%
      ## Nest by individual
      nest(-indv) %>%
      ## Estimate cumulative probability and estimate cutoff value for each individual
      mutate(fit = map(data, ~ predict(smooth.spline(cumsum(sort(.$wght_prob)),
                                        sort(.$wght_prob), spar = 0.1), 1 - odds)),
             wght_cutoff = map_dbl(fit, "y")) %>%
      ## Remove spline predictions & unnest dataframe
      select(-fit) %>%
      unnest() %>%
```

```r
    ## Reclassify cells as likely/unlikely based on cumulative prob
    mutate(wght_origin = ifelse(wght_prob > wght_cutoff, 1, 0))
  }

  return(wght_summ)
}
```

To estimate model performance, we modify the `error_rate` and `area_assign` functions to take the new `wght_origin` assignments and then measure the performance of each model:

```r
error_rate <- function(df, origin_cell, iso = TRUE){
  if(iso == TRUE){
    # Convert vector of likely/unlikely classifications to cell x indv matrix
      origin <- matrix(df$iso_origin, ncol = length(unique(df$indv)), byrow = FALSE)
    # Extract classification for the true origin of each individual
      correct <- origin[cbind(origin_cell, seq_along(origin_cell))]
    # Estimate error rate
      err_rate <- 1 - mean(correct)
  }else{
    origin <- matrix(df$wght_origin, ncol = length(unique(df$indv)), byrow = FALSE)
    correct <- origin[cbind(origin_cell, seq_along(origin_cell))]
    err_rate <- 1 - mean(correct)
  }
  return(err_rate)
}

area_assign <- function(df, iso = TRUE){
  #### Average # cells identified as likely for isotope only assignment
  if(iso == TRUE){
    area <- df %>%
      group_by(indv) %>%
      summarise(cells = sum(iso_origin), area = cells / length(iso_origin)) %>%
      .$area
  }else{
    area <- df %>%
      group_by(indv) %>%
      summarise(cells = sum(wght_origin), area = cells / length(wght_origin)) %>%
      .$area
  }
  return(mean(area))
}
```

```r
wght_area  <- plyr::ldply(wght_summ, function(x)
               data.frame(area = area_assign(df = x, iso = FALSE)))
wght_error <- plyr::ldply(wght_summ, function(x)
               data.frame(error = (error_rate(df = x,
                                   origin_cell = origin.cell, iso = FALSE))))
```

Pareto optimality

The final step of the analysis is to determine which of the 442 models provide reasonable solutions for assignment of unknown-origin birds. We do this using Pareto optimality, a multi-objective optimization

method most commonly used in economics and engineering. Briefly, Pareto optimality describes a situation where a change in the system (e.g. changing the abundance and/or isotope weights) cannot improve one performance metric without worsening the other. Pareto optimal models are said to "dominate" all other models because they have both lower assignment area and error rate than others in the model set. The subset of models that are Pareto optimal form the Pareto frontier, along which one cannot improve assignment error without increasing assignment area and vice versa. To apply this concept to our analysis, we first create a data frame that stores the performance metrics for all of the 442 models:

```
iso_perf <- data.frame(abun_weight = NA, iso_weight = 1,
                       area = iso_area, error = iso_error)

wght_perf <- data.frame(abun_weight = log(weights$iso_weight, base = 10),
                        iso_weight  =  log(weights$abun_weight, base = 10),
                        area = wght_area$area, error = wght_error$error)

mod_perf <-  bind_rows(iso_perf, wght_perf)
```

Next, we define a function to estimate the Pareto frontier and apply it to the model results:

```
pareto <- function(df) {
  df.sorted <- df[with(df, order(df$error, df$area)),]
  front <- df.sorted[which(!duplicated(cummin(df.sorted$area))),]
  return(front)
}
```

```
frontier <- pareto(mod_perf)
```

Because many of the models along the Pareto frontier may have very high error rates, the final step of the analysis is to subset only the Pareto optimal models that have a lower error rate and area than the isotope-only model:

```
top_assign <- function(front, df){
  baseline <- filter(df, is.na(abun_weight))
  error_base <- baseline$error
  area_base <- baseline$area


  top <- filter(front, error < error_base * error_mult  & area < area_base)
  return(top)
}
```

```
top <- top_assign(front = frontier, df = mod_perf)
```

Application: Assignment of Wood Thrush

Below, we demonstrate the complete assignment workflow using known-origin feather samples from Wood Thrush collected at 5 sites across the breeding range. In addition to the assignments, we include code to visualize both the assignment maps and the Pareto results.

Load and format the data:

```r
load("Data/WOTH_data.RData")
woth_dd$state <- toupper(woth_dd$state)
```

Isotope-only assignment:

```r
### Isotope-only model
woth_iso <- iso_assign(D = woth_d2H, B = woth.base$df.ahy, lat = woth.base$y,
                       lon = woth.base$x, odds = 0.67)
```

```r
# Estimate isotope-only summary stats
woth_iso_area  <- area_assign(summ = woth_iso)
woth_iso_error <- error_rate(summ = woth_iso, origin_cell = woth_origin_cell)

woth_iso_perf <- data.frame(abun_weight = NA, iso_weight = 0,
                            area = woth_iso_area, error = woth_iso_error)
```

Weighted assignment:

```r
woth_wght <- weight_assign(iso_assign = woth_iso, A = woth.base$rel.abun, min_weight = -1,
                           max_weight = 1, odds = 0.67)

# Estimate isotope-only summary stats
woth_area  <- plyr::ldply(woth_wght, function(x)
               data.frame(area = area_assign(summ = x, iso = FALSE)))
woth_error <- plyr::ldply(woth_wght, function(x)
               data.frame(error = error_rate(summ = x,
                          origin_cell = woth_origin_cell, iso = FALSE)))

woth_wght_perf <- data.frame(abun_weight = log(weights$abun_weight, base = 10),
                             iso_weight  =  log(weights$iso_weight, base = 10),
                             area = woth_area$area, error = woth_error$error)

woth_summ <-  dplyr::bind_rows(woth_iso_perf, woth_wght_perf)
```

Determine Pareto frontier and top models:

```r
woth_front <- pareto(df = woth_summ)
woth_top <- top_assign(front = woth_front, df = woth_summ)
```

Plot model performance and Pareto frontier:

```r
ggplot(woth_summ, aes(x = error, y = area)) +
  geom_point(color = "grey75") +
  geom_point(data = woth_front, color = "red") +
  geom_point(data = woth_top, color = "blue") +
  scale_y_continuous("Assignment area (%)") +
  scale_x_continuous("Error rate (%)")
```

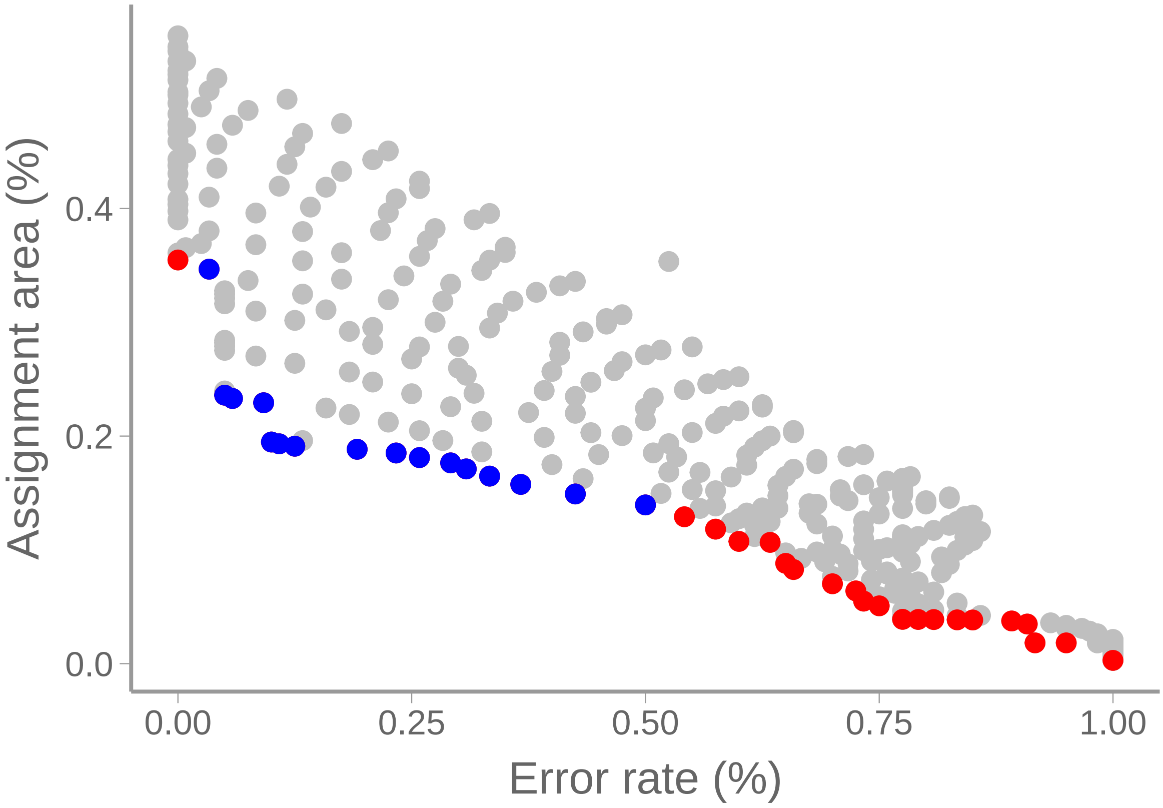

Map of relative abundance and sampling locations:

```
woth.abun  <- ggplot() +
  geom_tile(data = woth.base, aes(x = x, y = y, fill = rel.abun, color = rel.abun)) +
  scale_fill_gradient(name = "Rel. abun.", low = "#ffeda0", high = "#bd0026") +
  scale_color_gradient(low = "#ffeda0", high = "#bd0026") +
  scale_y_continuous(limits = c(25, 54)) +
  scale_x_continuous(limits = c(-108, -63)) +
  geom_polygon(data=woth.states, aes(x=long, y=lat, group = group),
               colour="black", fill = NA) +
  coord_map("lambert", lat0 = 40, lat1 = 20) +
  labs(title = "Wood Thrush relative abundance") +
  theme(axis.line=element_blank(),axis.text.x=element_blank(),
        axis.text.y=element_blank(),axis.ticks=element_blank(),
        axis.title.x=element_blank(),
        axis.title.y=element_blank(), panel.border = element_blank(),
        plot.margin = unit(c(1, 1, 1, 1), "mm"),
        legend.text = element_text(size = 14),
        legend.title = element_text(size = 16)) +
  guides(color = FALSE) +
  geom_point(data = woth.sta, aes(x = x, y = y), size = 4.5, color = "black", shape = 17)

woth.abun
```

```
## Warning: Removed 8 rows containing missing values (geom_tile).
```

Wood Thrush relative abundance

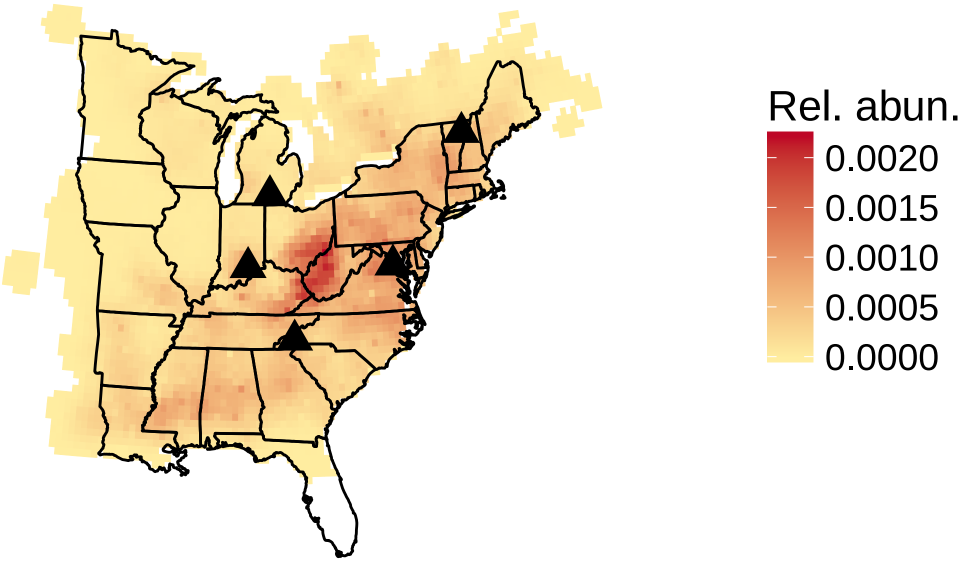

Map isotope-only assignment results for one individual:

```
woth.1  <- woth_iso %>%
  filter(indv == "Indv_1") %>%
  ggplot(data = .) +
  geom_tile(aes(x = lon, y = lat, fill = iso_prob, color = iso_prob)) +
  scale_fill_gradient(name = "Post. prob.", low = "#ffeda0", high = "#bd0026") +
  scale_color_gradient(low = "#ffeda0", high = "#bd0026") +
  scale_y_continuous(limits = c(25, 54)) +
  scale_x_continuous(limits = c(-108, -63)) +
  geom_point(data = data.frame(x = -85.82, y = 38.83), aes(x = x, y = y),
             size = 4.5, shape = 17) +
  geom_polygon(data=woth.states, aes(x=long, y=lat, group = group),
               colour = "black", fill = NA) +
  coord_map("lambert", lat0 = 40, lat1 = 20) +
  theme(axis.line=element_blank(),axis.text.x=element_blank(),
        axis.text.y=element_blank(),axis.ticks=element_blank(),
        axis.title.x=element_blank(),
        axis.title.y=element_blank(), panel.border = element_blank(),
        plot.margin = unit(c(1, 1, 1, 1), "mm"),legend.text = element_text(size = 14),
        legend.title = element_text(size = 16)) +
  guides(color = FALSE)

woth.1
```

```
## Warning: Removed 8 rows containing missing values (geom_tile).
```

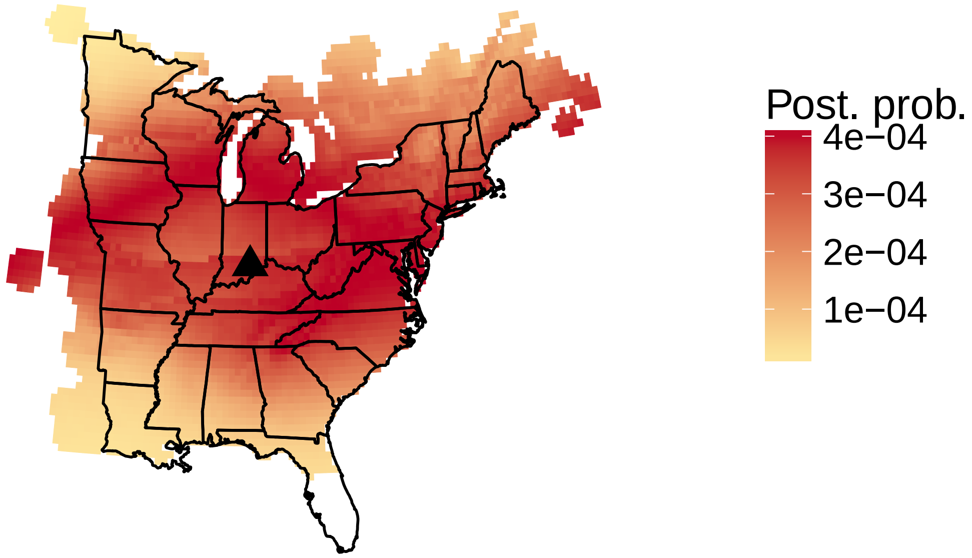
Post. prob.
4e−04
3e−04
2e−04
1e−04
